# Fast and Reliable Electronic Assay of a *Xylella fastidiosa* Single Bacterium in Infected Plants Sap

**DOI:** 10.1002/advs.202203900

**Published:** 2022-08-28

**Authors:** Lucia Sarcina, Eleonora Macchia, Giuliana Loconsole, Giusy D'Attoma, Paolo Bollella, Michele Catacchio, Francesco Leonetti, Cinzia Di Franco, Vito Elicio, Gaetano Scamarcio, Gerardo Palazzo, Donato Boscia, Pasquale Saldarelli, Luisa Torsi

**Affiliations:** ^1^ Dipartimento di Chimica Università degli Studi di Bari “Aldo Moro” Bari 70125 Italy; ^2^ Dipartimento di Farmacia – Scienze del Farmaco Università degli Studi di Bari “Aldo Moro” Bari 70125 Italy; ^3^ Institute for Sustainable Plant Protection CNR Bari 70125 Italy; ^4^ Istituto di Fotonica e Nanotecnologie CNR c/o Dipartimento Interateneo di Fisica Università degli Studi di Bari Aldo Moro Bari 70125 Italy; ^5^ Agritest Srl Tecnopolis Casamassima BA 70010 Italy; ^6^ Dipartimento Interateneo di Fisica Università degli Studi di Bari Aldo Moro Bari 70125 Italy

**Keywords:** electrolyte gate organic field‐effect transistor, single‐molecule assay with a large transistor, single bacterium sensing

## Abstract

Pathogens ultra‐sensitive detection is vital for early diagnosis and provision of restraining actions and/or treatments. Among plant pathogens, *Xylella fastidiosa* is among the most threatening as it can infect hundreds of plant species worldwide with consequences on agriculture and the environment. An electrolyte‐gated transistor is here demonstrated to detect *X. fastidiosa* at a limit‐of‐quantification (LOQ) of 2 ± 1 bacteria in 0.1 mL (20 colony‐forming‐unit per mL). The assay is carried out with a millimeter‐wide gate functionalized with *Xylella*‐capturing antibodies directly in saps recovered from naturally infected plants. The proposed platform is benchmarked against the quantitave polymerase chain reaction (qPCR) gold standard, whose LOQ turns out to be at least one order of magnitude higher. Furthermore, the assay selectivity is proven against the *Paraburkholderia phytofirmans* bacterium (negative‐control experiment). The proposed label‐free, fast (30 min), and precise (false‐negatives, false‐positives below 1%) electronic assay, lays the ground for an ultra‐high performing immunometric point‐of‐care platform potentially enabling large‐scale screening of asymptomatic plants.

## Graphical abstract

An electrolyte‐gated transistor is demonstrated here to detect *Xylella fastidiosa* at a limit‐of‐quantification of 2 ± 1 bacteria in 0.1 mL (20 colony‐forming‐unit per mL). The proposed label‐free, fast (30 min), and precise (false‐negatives, false‐positives below 1%) electronic assay lays the ground for an ultra‐high performing immunometric point‐of‐care platform potentially enabling large‐scale screening of asymptomatic plants.

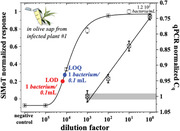

## 1 Introduction

Studies on a single‐bacterium are relevant as, even in clonal populations, bacterial cell‐to‐cell variability at the microbiological (metabolism profile, response to stimuli, survival ability,^[^ ^1^, ^2^, ^3^, ^4^, ^5^, ^6^, ^7^ ^]^ etc.) and biochemical level, can be significant.^[^ ^8^ ^]^ However, bacteria cells, being two–three orders of magnitude smaller than eucaryote ones, cannot be easily investigated as single entities. A surge of technologies that can scrutiny an ensemble of bacteria at a single‐cell resolution has, hence, appeared.^[^ ^8^ ^]^ These platforms largely rely on microfluidic‐based approaches involving wells with ultra‐small volumes (10^−12^–10^−9^ L) where a bacterium is trapped^[^ ^9^, ^10^, ^11^, ^12^ ^]^ and investigated with near‐field approaches.^[^ ^13^, ^14^ ^]^ Single‐bacterium detection in a real sample, necessarily involves much larger volumes (e.g., 10^−5^–10^−4^ L); hence, near‐field probing, involving the time‐consuming serial inspection of 10^5^–10^7^ sample portions,^[^ ^15^ ^]^ becomes inapplicable. In the specific case of the *Xylella fastidiosa* bacterium, plate‐counting by means of culture‐based methods is also time‐consuming as this is a slow‐growing cell. Moreover, sample handling can be laborious and not suited for point‐of‐care in‐fields, fast assays.^[^ ^16^ ^]^ The so far proposed single‐bacterium detecting technologies are generally also label‐needing,^[^ ^15^, ^16^ ^]^ which further increases samples’ processing time. No wonder, ultra‐sensitive bacteria assays carried out in a real biofluid, have been only very seldom proposed.^[^ ^15^ ^]^

Point‐of‐care detection of infectious diseases can be very relevant for rapid screening and early diagnosis at low‐cost.^[^ ^17^ ^]^ Indeed, with a fast, reliable, and portable detecting system, large screening of the asymptomatic plants becomes possible and can effectively reduce an infection spreading along with its burden on society, ecosystems, and economy.^[^ ^17^, ^18^ ^]^ They preferably require a label‐free approach as it is shown in the CMOS‐array capacitive bacteria detection,^[^ ^19^ ^]^ but detections were not carried out in a real fluid. The Single‐Molecule assay with a large‐Transistor (SiMoT) technology encompasses an electrolyte‐gated organic field‐effect‐transistor (EGOFET)^[^ ^5^ ^]^ endowed with a wide electrode interface and has been lately proven to perform immunoassay of protein markers at limit‐of‐detection (LOD)^[^ ^6^ ^]^ of 10^−20^ mole L^−1[^ ^20^ ^]^ so that, in 0.1 mL, 1 ± 1 molecule was detected.^[^ ^21^ ^]^

Among plant bacteria, *X. fastidiosa* (a gram‐negative, rod‐shaped bacterium within the Xanthomonadaceae family) is one of the most threatening, as it can infect more than 600 plant species^[^ ^1^ ^]^ along with several crops, with large economic losses. After its first outbreak and identification in Apulia in 2013,^[^ ^22^ ^]^ *X. fastidiosa* has been steadily spreading and it is now threatening the Mediterranean olive agriculture area, one of the largest worldwide. So far the surveillance measures involve diagnostic tools such as quantitative real‐time polymerase chain reaction (qPCR),^[^ ^23^, ^24^ ^]^ or loop‐mediated isothermal amplification.^[^ ^7^ ^]^ Lately also, a surface‐plasmon resonance label‐free approach has been successfully undertaken.^[^ ^25^ ^]^ These approaches are based on bench‐top instrumentations, hardly prone for fast and convenient in‐field detections. Moreover, while qPCR‐based techniques can reach limit‐of‐quantifications (LOQs) of 10^2^ colony‐forming‐unit (cfu) per mL,^[^ ^24^ ^]^ all the other assays can, at the very best, detect 10^4^–10^5^ cfu mL^−1^.

The present work deals with a SiMoT device capable of electronic label‐free detection of a single‐bacterium of *X. fastidiosa* in 0.1 mL with high precision and reliability as false‐negative and false‐positive errors are lower than 1%. This is accomplished by means of a millimeter‐squared wide gate‐electrode covered by 10^11^ anti‐*X. fastidiosa* (anti‐*Xf*) recognition antibodies. The assay is carried out by exposing the biofunctionalized gate directly to field samples whose bacteria content is benchmarked against qPCR.^[^ ^7^ ^]^ The latter is proven to reach LOQs at least one order of magnitude higher. Negative control experiments, involving the anti‐*Xf* covered gate, exposed to the non‐target gram‐negative bacterium *Paraburkholderia phytofirmans*, PsJN, prove that the assay is also highly selective. Moreover, the overall assay time to results is as low as 30 min.

## 2 Results and Discussion

**Figure 1:**
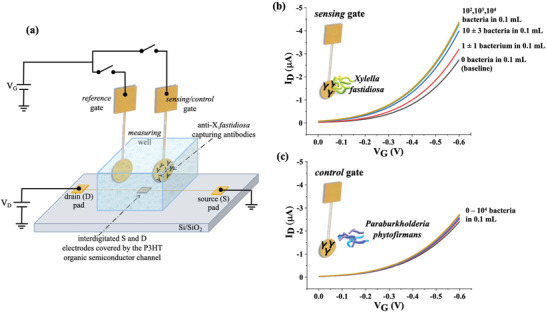
The SiMoT sensing device and its output curves: a) The electrolyte‐gated organic field‐effect‐transistor (EGOFET) device structure on an insulating substrate with interdigitated source (S) and drain (D) electrodes forming a channel with a width/length ratio of 5 µm/10^4^ µm that are connected to larger pads. Poly(3‐hexylthiophene‐2,5‐diyl)—P3HT, serves as the organic semiconducting layer. A measuring well, filled with 0.3 mL of deionized water, is sealed around the FET channel area leaving the source and drain pads outside. The bare gold reference gate is immersed in this well too where it stays fixed throughout the assay. The sensing/control gate biofunctionalized with the capturing anti‐*X*. *fastidiosa* (anti‐*Xf*) antibody layer is also immersed in the measuring well. Here, the gate work function shift that occurred in the incubation well upon affinity binding of the *X*. *fastidiosa* bacterium to its capturing antibody, is assessed as a source–drain *I*_D_ current change in the EGOFET channel. The incubation of the sensing/control gate in the sample to be assayed is accomplished by immersing the gate into the incubation well (not shown) for 10 min. b) Transfer characteristics (source–drain current *I* vs gate bias *V*_G_ at a fixed drain bias of −0.3 V) were measured with the sensing gate assaying the *Xylella* bacterium. The black curve was measured after nurturing the sensing gate in 0.1 mL of a PBS solution (ionic strength = 162 mm, pH = 7.4) for 10 min; afterward, the gate was washed thoroughly in HPLC‐grade water (ionic strength ≈5 µm and pH = 5.5) and immersed in the measuring well. Subsequently, the very same gate was immersed for 10 min in 0.1 mL of a PBS solution added (spiked) with *X. fastidiosa* bacteria resulting in a 10 cfu mL^−1^ solution. Assuming that one infecting bacterium forms one cfu, in the assayed volume there is 1 ± 1 bacterium. The measured curve is the red one. The blue curve corresponds to the assay of 10 ± 3 bacteria in 0.1 mL PBS, while the green, violet, and yellow are relevant to 10^2^, 10^3^, and 10^4^ bacteria in 0.1 mL PBS solutions, respectively. The reference gate is coupled to the OFET channel before measuring the baseline and after the measurement of the last concentration. The resulting currents are compared, and the sensing experiment is retained only if their relative fractional change is lower than 5–8%. c) Transfer characteristics were measured on the anti‐*Xf* functionalized electrode (control gate) assaying the non‐target *P. phytofirmans* bacterium (PsJN) at the same concentrations as in panel (b).

In **Figure** **1**a the SiMoT device structure comprising the sensing*/*control and the reference gates as well as the transistor channel is shown. The channel encompasses the source (S) and drain (D) electrodes covered by the p‐type organic semiconductor. The sensing*/*control gate is biofunctionalized, as detailed in the Experimental Section, with trillions of anti‐*Xf* antibodies that highly specifically bind to the bacterium. This takes place when the gate is nurtured for 10 min in the incubation well (not shown) containing 0.1 mL of the field samples or the standard solutions to be assayed. There is in fact a chance larger than 70% that a diffusing bacterium hits a large sensing gate in 0.1 mL, during the 10 min of incubation^[^ ^26^ ^]^ (vide infra). Upon binding, the sensing gate work function sizably shifts, and this is measured by capacitively coupling the gate to the transistor channel whose current changes, in turn.^[^ ^21^ ^]^ To maximize the output electronic sensing response it is necessary to maximize the Debye's length. The samples to be assayed are high ionic strength (162 mm) media and so the sensing gate is assessed in the measuring well filled with low ionic strength (≈5 µm) deionized (DI) water.^[^ ^21^ ^]^ The reference gate, stably hanging over the channel, enables to control the device stability during the whole assay. In Figure 1b, the transistor output transfer curves measured with the sensing gate assaying *Xylella*, are shown. The black curve is the baseline, *I*_0_, measured with the biofunctionalized gate incubated for 10 min in a bare PBS (ionic strength = 162 mm) solution. It is then washed with DI water and positioned in the measuring well, filled also with DI water (ionic strength ≈5 µm). The same sensing gate is incubated, afterward, in 0.1 mL of PBS added with the *X*. *fastidiosa* bacteria (10 cfu mL^−1^). Assuming that one bacterium forms 1 cfu, in the assayed volume of 0.1 mL of a 10 cfu mL^−1^ solution, there is 1 ± 1 bacterium, the indetermination being the Poisson sampling error.^[^ ^21^ ^]^ Eventually, the red curve, *I*, is measured in water after the binding of a single bacterium. The other curves in Figure 1b are measured after nurturing the same sensing gate in the PBS solutions with 10, 10^2^, 10^3^, and 10^4^ bacteria in 0.1 mL. The maximum relative shift of the measured current (*I*), compared to the baseline (*I*_0_), namely (−*ΔI/I*_0_) = [−(*I* − *I*_0_)/*I*_0_], at the maximum transconductance, *(−ΔI/I*_0_)^max^, is as high as 0.68 ± 0.01 (error taken as one standard deviation). In Figure 1c the negative control assay is shown, involving anti‐*Xf* functionalized gate analyzing a PBS solution added (spiked) with the non‐target gram‐negative bacterium PsJN. In this case, the 0–10^4^ bacteria in 0.1 mL range, was covered and the maximum relative shift *I*, compared to the baseline, *I*_0_, at the maximum transconductance, *(−ΔI/I*_0_)^min^, is 0.07 ± 0.01. As an assay validation tool, the reference gate was coupled to the channel before starting and after completing the whole assay, resulting in a maximum current relative shift below 0.05–0.08 so within the overall change of the negative control experiment (vide infra).

**Figure 2:**
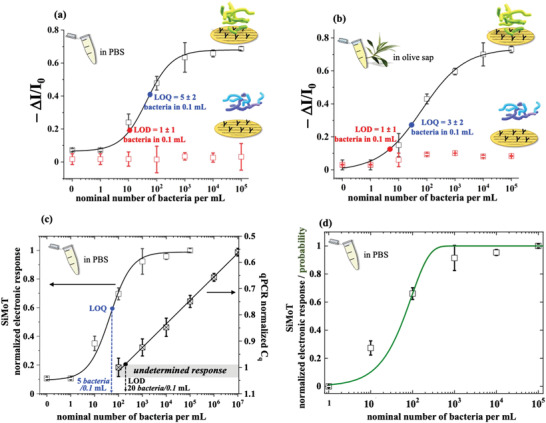
SiMoT assays in PBS and in olive sap solutions spiked with *X*. *fastidiosa* and PsJN bacteria, benchmarked toward qPCR: a) Dose curves of the *X. fastidiosa* electronic SiMoT assay carried out in PBS standard solutions with concentrations in the 0–10^5^ bacteria per mL range. The electronic response is taken as the negative of the relative current shift (*I*) with the respect to the baseline *I*_0_ (−*ΔI*/*I*_0_). The black hollow squares are the data measured with the sensing gate in the *Xylella*‐containing samples, while the red hallow squares are those measured in the negative control experiment that assays the non‐target PsJN. Error bars over three replicates are set as one standard deviation. The modeling (black solid curve) has been performed with an analytical model based on a 4‐parameter logistic equation (see Experimental Section), whose four parameters are: (−*ΔI*/*I*_0_)^max^ = 0.68 ± 0.01, (−*ΔI*/*I*_0_)^min^ = 0.07 ± 0.01, *x*_0_ = 44 ± 14 bacteria per mL and a *p* = 0.97 ± 0.2, leading to an average distribution of the number of anti‐*Xf* in each domain of $\bar{x}$ = 4.2 × 10^10^. The LOD, taken as the average of the control experiment data (noise level) plus three times the standard deviation, is equal to 1 ± 1 bacterium in 0.1 mL (10 cfu/mL or equivalently 10 bacteria per mL). The LOQ (by the definition the level of the average of the control experiment data plus nine times the standard deviation) is equal to 5 ± 2 bacteria in 0.1 mL (54 bacteria per mL). b) Dose curves of the *X. fastidiosa* electronic SiMoT assay carried out in 0.1 mL olive sap from *Xylella*‐free olive sources, added with bacteria to spam concentrations in the 0–10^5^ bacteria per mL range. The modeling (solid black curve) has been performed with an analytical model based on a 4‐Parameter logistic whose parameters are: (−*ΔI/I*_0_)^max^ = 0.74 ± 0.02, (−*ΔI/I*_0_)^min^ = 0.01 ± 0.03, *x_0_* = 67 ± 21 bacteria per mL and *p* = 0.97 ± 0.1. The average of the distribution of the number of anti‐*Xf* comprised in each domain for the bacteria assay in sap, is as high as $\bar{x}$ = 6.5 × 10^10^. LODs and LOQs in the saps’ assay are: LOD = 7 bacteria per mL and LOQ = 33 bacteria per mL. c) A comparison of the SiMoT and the qPCR dose curves was carried out on the same *X*. *fastidiosa* standard solutions in PBS in the 0–10^7^ bacteria per mL range. Also in this case the data are relevant to three replicates and the errors are taken as one standard deviation. The values in the abscissa are: on the left the −Δ*I*/*I*_0_ data of panel (a) normalized by the SiMoT response saturated value (−*ΔI*/*I*_0_)^max^ = 0.68; on the right, the qPCR values of the quantification cycle, *C*_q_, normalized by the highest number of cycles, *C*_q_^max^ = 33.64 cycles, needed to assay the sample with the lowest concentration. The qPCR responses below 200 bacteria per mL (gray shaded area) are those categorized as undetermined samples, ascribed neither to the positive nor to the negative response. This marks the level of the background fluorescence plus nine times noise’ standard deviation (see Experimental Section) hence being the LOQ level. d) Modeling of the normalized SiMoT dose curve in PBS with the probability function^[^ ^28^ ^]^ computes what is the probability that one bacterium, out of those present in the 0.1 volume, hits the 0.2 cm^2^ biofunctionalized electrode surface. See the Experimental Section for details.

In **Figure** **2**a the data introduced in Figure 1, are plotted as dose curves of the (−*ΔI/I*_0_) SiMoT electronic response for both the sensing (black squares) and the control (red squares) experiments carried out in *X*. *fastidiosa* and PsJN spiked PBS solutions. The modeling of the SiMoT sensing response (solid black curve) was carried out by means of an analytical model based on Poisson distribution probability,^[^ ^21^ ^]^ encompassing a 4 parameters logistic equation. This accounts for the occurrence of a few binding events (see Experimental Section). The negative control experiment gives the noise level in the assay, being (−*ΔI/I*_0_) = 0.09 ± 0.04. The LOD level, taken as the concentration corresponding to the noise average level plus three times its standard deviation,^[^ ^6^ ^]^ is as low as 10 cfu mL^−1^ while the LOQ (noise average level plus nine times the standard deviation)^[^ ^6^ ^]^ is 54 cfu mL^−1^. The sampled solutions are all 0.1 mL hence the LOD and the LOQ correspond to 1 ± 1 and 5 ± 2 bacteria, respectively. This confirms, with a confidence level of 99.73% at the LOD and better than 99.9% at the LOQ, that the SiMoT assay is indeed able to detect a single bacterium in a typical real sample volume of 0.1 mL. In Figure 2b the same experiments are carried out in saps from *Xylella*‐free olive sources, added with the *Xylella* or the PsJN bacteria. In this very complex real fluid, the level of the average noise (red squares) is comparable to what is measured in PBS, being 0.08 ± 0.03. The LOD and LOQ levels are 7 and 33 bacteria per mL, respectively, which returns single‐bacterium detection capabilities of the SiMoT platform in 0.1 mL of olive sap as well. In Figure 2c a systematic comparison of the SiMoT and qPCR *X. fastidiosa* assays in PBS is provided. The responses (electronic output on the left *Y*‐axis and quantification cycles on the right *Y*‐axis) are normalized to the respective highest values while the concentration spans the 0 (bare PBS)–10^7^ bacteria per mL range. For the qPCR, the LOQ is 20 bacteria per 0.1 mL. In Figure 2d the SiMoT normalized responses are modeled with a probability function based on the Einstein diffusion equation, specifically designed to model the SiMoT assay dose curves.^[^ ^26^ ^]^ A very good agreement between the data and the model, using only the bacterium diffusion constant as a parameter is provided. Moreover, the diffusion constant value extracted is 3.3 µm^2^ s^−1^ which is perfectly in line with the free diffusion constant of a bacterium in a bulk liquid falling in the 2.0–7.9 µm^2^ s^−1^ range.^[^ ^27^ ^]^ This implies that a single bacterium (out of a few) acting as a Browning particle obeying Einstein's diffusion theory, can strike, with a very high probability, a large‐area gate functionalized with 10^11^ antibodies, within a few minutes.

**Figure 3:**
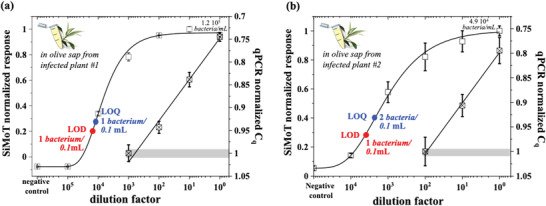
Field samples from *X. fastidiosa* infected plants assayed by SiMoT and qPCR: The SiMoT and the qPCR dose curves measured on the same *X*. *fastidiosa* field samples from naturally infected olive trees located in the contaminated area of Apulia (southern Italy). a) The sap from infected plant #1 (maximum concentration assessed by qPCR: 1.2 × 10^5^ bacteria per mL). b) Sap from infected plant #2 (maximum concentration assessed by qPCR: 4.9 × 10^4^ bacteria per mL). The other points are relevant to tenfold standard dilutions in the sap recovered from a *Xylella*‐free olive source. The data are relevant to three replicates and the errors are taken as one standard deviation. The values in the abscissa are: on the left the (−Δ*I/I*_0_) data normalized by the SiMoT response saturated value (−Δ*I/I*_0_)^max^ of 0.95 (a) and of 0.93 (b); on the right, the qPCR values of the quantification cycle, *C*_q_, normalized by the highest number of cycles, *C_q_* ^max^ = 33.64 cycles, needed to assay the sample with the lowest concentration. The qPCR responses below 200 bacteria per mL (gray shaded area) are those that can be ascribed neither to the positive nor to the negative response. Also in this case the modeling (black solid curve) has been performed with an analytical model based on a 4‐parameter logistic equation (see Experimental Section) based on the same set of parameters derived from the calibration dose curve in sap.

In **Figure** **3**a,b, comparisons of the SiMoT and qPCR assays carried out on field samples from two different naturally infected olive trees, are given. In this case, the originally sampled saps were assayed by qPCR (see Experimental Section) and from these, tenfold standard diluted solutions in sap from *Xylella*‐free olive sources, were produced. Twins 0.1 mL samples were assayed at each dilution and the normalized *(−ΔI/I*_0_) and *C*_q_ data are plotted. The noise levels, computed as the average response of the biofunctionalized gate assaying *Xylella*‐free saps, resulted in a *(−ΔI/I*_0_) of 0.19 and 0.05 for plant #1 and plant #2, respectively. This leads to a LOD and LOQ of 1 ± 1 in 0.1 mL for plant #1. For plant #2 the LOD is 1 ± 1 in 0.1 mL while the LOQ is 2 ± 1 bacteria. Also in this case the modeling of the curves has been performed using a 4‐parameter logistic equation, based on the same set of parameters derived from the calibration dose curve in spiked sap, which very well describes the experimental data. The LOQ for the qPCR, in these cases, are 12 bacteria per 0.1 mL and 49 bacteria per 0.1 mL, respectively. Hence the SiMoT technology offers an improvement over qPCR better than a factor of 10 and 20, respectively.

Remarkably, the time‐to‐result of the SiMoT technology is of about 30 min per assay. Indeed, the assay foresees a first incubation step of the sensing gate of about 10 min in the reference fluid. The gate is then washed in DI water and the baseline level is then acquired. This latter step encompasses the acquisition of 20 subsequent transfer characteristics, requiring about 5 min. The sensing gate can be therefore incubated for 10 min in the plant sap to be assayed. A further washing step is foreseen before the signal is registered, cycling the SiMoT device with 20 subsequent transfer characteristics. In contrast, qPCR technology requires time‐to‐results of at least 3 h, since multi‐steps are needed to perform the assay. Moreover, the assay is carried out taking the LOQ as the threshold, which means that the platform is also highly precise, allowing a maximum incidence, for both false‐positive and false‐negative as well as for the *γ*‐type‐II error (overlap between the LOQ and the LOD) of less than 1%.^[^ ^6^ ^]^ This implies that in a qualitative assay, SiMoT is able to reliably assess (random error lower than 1%) if a sample is totally free from any *X. fastidiosa* bacterium or not. This is of high relevance in a screening campaign of asymptomatic plants as it enables to reliably isolate the subset of not infected samples.

## 3 Conclusion

In conclusion, a SiMoT device capable of electronic label‐free detection of *X. fastidiosa* gram‐negative filament bacterium at a LOQ of 2 ± 1 bacteria in 0.1 mL (20 bacteria per mL), is demonstrated. This is performed by means of a millimeter‐wide (0.2 cm^2^) gate bearing a layer of 10^11^ anti‐*Xf* recognition antibodies. The transducing gate is incubated for 10 min directly into 0.1 mL of a real sample whose bacterium content is independently assayed by qPCR.^[^ ^7^ ^]^ The latter is proven to offer detection limits at least tenfold better. Negative control experiments involve the anti‐*Xf* covered gate exposed to olive saps added with the non‐binding *P. phytofirmans* bacterium. The assay proposed, that is fast (time‐to‐results: 30 min) and accurate (errors below 1%), opens to ultra‐high performing immunometric point‐of‐care devices for large‐scale screening of asymptomatic plants.

## 4 Experimental Section

### Materials

Poly(3‐hexylthiophene‐2,5‐diyl) (P3HT, regioregularity > 99%, average molecular weight = 17.5 kDa), 3‐mercaptopropionic acid (3‐MPA) and 11‐mercaptoundecanoic acid (11‐MUA), 1‐ethyl‐3‐(3‐dimethyl aminopropyl)‐carbodiimide (EDC), *N*‐hydroxysulfosuccinimide sodium salt (sulfo‐NHS), K4[Fe(CN)6]·3H2O (98.5%), ethanol (grade puriss. p.a. assay ≥ 99.8%), and water (HPLC‐grade), were all purchased from Sigma–Aldrich and used with no further purification. Potassium chloride (Fluka, puriss p.a.) was also used with no further purification. Purified‐IgG against *X. fastidiosa* (analytical specificity validated through EPPO standard^[^ ^24^ ^]^) anti‐*Xf* at a concentration of 1 mg mL^−1^ were provided by Agritest and diluted to 10 µg mL^−1^.

### Xylella fastidiosa and Paraburkholderia phytofirmans, PsJN, Solutions in PBS and Olive Sap

10 days‐old colonies of *X. fastidiosa* (gram‐negative) subsp. pauca De Donno strain, sequence‐type ST53 and 2 days‐old colonies of *P. phytofirmans* PsJN (gram‐negative, rod‐shaped bacterium) were scraped from plates and dispersed in sterile phosphate buffer saline—PBS (50 mm, pH 7.2). These *Xylella* and PsJN mother solutions were spiked in a more concentrated PBS (162 mm and pH 7.4) solution or in olive sap from *Xylella*‐free olive sources. The sap was recovered by homogenizing 0.5 g of ≈1 cm long shoots in PBS, and further centrifugated (8000 × g for 1 min). The supernatant sap was finally diluted 1:4 in PBS (162 mm and pH 7.4). Tenfold diluted solutions were prepared by the standard dilution method. The effective bacteria concentrations of the *Xylella* and PsJN solutions (both in PBS or in olive sap), expressed in cfu mL^−1^, were determined by plate counting.^[^ ^29^ ^]^ According to EPPO protocols, it is assumed that 1 cfu corresponds to a single bacterium.^[^ ^24^ ^]^

### Samples from Infected Plants

The saps from two symptomatic naturally infected olive trees located in the infected area of Apulia (southern Italy), were recovered as previously described. The infected saps were diluted (by the standard tenfold dilution method) in the sap recovered from a *Xylella*‐free olive source. The bacterium concentration in each sample was quantified by qPCR assay^[^ ^30^ ^]^ on DNA extracts, obtained by Maxwell RSC PureFood GMO and Authentication Kit (Promega).^[^ ^31^ ^]^ The same samples were also analyzed by the SiMoT technology.

### Organic Electrolyte‐Gated FET Fabrication

The EGOFETs were fabricated, as fully detailed elsewhere,^[^ ^21^, ^32^, ^33^ ^]^ starting from a Si/SiO_2_ substrate patterned with photo‐lithographically defined source (S) and drain (D) interdigitated electrodes deposited by e‐beam evaporation of Ti(5 nm) and Au(50 nm) tacking layers. The channel length of 5 µm and the width of 10^4^ µm defined an effective channel area of 5.3 × 10^−2^ mm^2^. A solution of P3HT (2.6 mg ml^−1^ in C_6_H_5_Cl), was spin‐coated (2 × 10^3^ rpm for 20s) on the electrodes’ area and was annealed afterward at 90 °C for 15 s. A polyurethane well was glued around the channel area and filled with the gating medium (0.3 mL of deionized water HPLC‐grade). Two circular electrodes (≈0.2 cm^2^) serving as reference and sensing or the control gates (G) were fabricated by e‐beam evaporation (Ti/Au, 5/50 nm) on a PEN foil. The gates serving as sensing or control electrodes were biofunctionalized (vide infra) with the anti‐*Xf* antibodies. The reference gate was not biofunctionalized and allowed the measurement of the current flowing in the channel at every stage of the assay.

### Gate Biofunctionalization Protocol

The sensing gate electrode was biofunctionalized following a protocol published elsewhere.^[^ ^21^, ^25^, ^32^, ^34^ ^]^ In the following the salient information is given. The biofunctionalization started with a 3‐MPA and 11‐MUA mixed chemical self‐assembled monolayer (SAM) activated with EDC‐sulfoNHS chemistry. The anti‐*Xf* antibodies were covalently attached to the activated SAM by immersion in a 10 µg mL^−1^ antibodies solution for 3 h. The activated carboxylic groups that remained unreacted, were processed in ethanolamine to become deactivated. A multiparameter surface plasmon resonance study proved that this protocol enabled to reach a very highly dense coverage of capturing anti*‐Xf* of 3.6 ± 0.8 × 10^11^ molecules cm^−2^.^[^ ^25^ ^]^

### Diffusion of a Single Bacterium at a Millimeter‐Wide Capturing Surface

The free diffusion constant of a bacterium in a bulk liquid is *D* = 200 − 1900 µm^2^ s^−1^.^[^ ^27^, ^35^ ^]^ This implied, according to Einstein equation *Δr* = (6 *D* × *Δt*)^1/2^, that in a *Δt* of 600s, the bacterium spanned on average a sphere with a radius *Δr* of about 0.8–2.6 mm. A probability function, specifically designed for the SiMoT platform (see reference^[^ ^26^ ^]^ for details) was used to model the normalized SiMoT response for the detection of the *X*. *fastidiosa* bacterium in PBS. Under these circumstances, there was a probability of ≈70%, that at least one bacterium out of 10 in 0.1 mL hits the large sensing gate (about 0.2 cm^2^) being hence captured and detected. While 10 min of incubation time was conveniently low and had resulted in a set of very reliable data,^[^ ^36^ ^]^ to enhance the probability of binding^[^ ^26^ ^]^ an optimized incubation time will be systematically investigated.

### SiMoT Sensing Measurements

The capacitive coupling between the gate and the transistor channel allowed the measurement of a source–drain current shift upon sensing.^[^ ^5^, ^21^, ^37^ ^]^ To be sure that the current in the transistor channel was stable throughout the sensing assay, the reference gate was always kept in the measuring well. The biofunctionalized sensing/control gates were nurtured in the incubation well, filled with the *Xylella* solutions to be analyzed, and assessed in the measurement well. For the *s*ensing gate, the fluid was PBS or olive sap was added with the *X. fastidiosa* bacterium while for the control *gate* the sample was added with the *P. phytofirmans*. The sensing measurements protocol, fully detailed elsewhere,^[^ ^38^ ^]^ involved at first the stabilization of the current in the FET channel via the reiterated measurements of the EGOFET current–voltage transfer curve (*I* vs the gate bias at a fixed source–drain bias of −0.3 V) in the measuring well using the reference electrode as the gate. The measurement runs until a stable current was measured for three subsequent cycles (stabilizing cycling). The sensing/control gate was nurtured for 10 min in the incubation well filled with 0.1 mL of bare PBS or olive sap (at RT and in the dark). Afterward, the sensing gate was extensively washed with DI (HPLC) water, it was transferred to the measuring well and a new cycle of repeated transfer characteristics was registered. After 20 cycles, when a stable *I*_0_ baseline is measured, the same sensing gate was removed from the measuring well and transferred back into the incubation well filled with a 0.1 mL of PBS or olive sap‐based solutions of *X. fastidiosa* (sensing) or of *P. phytofirmans* (control). The currents measured after incubation in each *Xylella* solution were addressed as the “*I*” signal at a given concentration. The (−*ΔI/I*_0_) = [−(*I* − *I*_0_)/*I*_0_] was taken as the electronic response and the relevant curves were obtained by plotting the data at the gate‐bias value that maximized the trans‐conductance *δI/δV*_D_ (falling generally for *V*_D_ in the −0.3 to −0.4 V range). All the data points were averaged over three replicates and the reproducibility error was computed as one standard deviation. The reference gate was coupled to the OFET channel before sensing the baseline and after the measurement of the last concentration. The resulting currents were compared, and the sensing experiment was retained only if their relative fractional change was lower than 5–8%. The LOD was computed by taking the average signal of the negative control experiment and adding its tree sigma values to it. In this case, the incidence of false positives was below 1% while that of false‐negatives was up to 50%. The LOQ was computed by taking the average signal of the negative control experiment and adding its nine sigma values to it. In this case, the incidence of false‐positives and false‐negatives as well as the *γ*—type‐II error, namely the overlap between the LOQ and the LOD, of less than 1%.^[^ ^6^ ^]^

The whole study here presented involved 81 assays (each data point in the three figures was taken in triplicates) that were measured in real infected samples as well as in spiked phosphate buffer solution or healthy pants’ sup. To these data, other 81 assays add up which included all the Paraburkholderia bacterium negative‐control experiments and the data collected for the benchmark against the qPCR.

### Modeling of the SiMoT Sensing Dose Curves

The black solid curves in Figure 2a,b were the fitting of the dose curves in PBS and sap solutions of *Xylella fastidiosa*, with an analytical function based on the Poisson distribution probability to better account for the occurrence of few binding events. Each data point in a given *ΔI/I*_0_ dose curve had been assumed to be proportional to the number of domains in which the gate work‐function had been switched by the binding with at least one bacterium, as described elsewhere.^[^ ^37^ ^]^ Each domain comprised a given number of capturing anti‐*Xf*, was characterized by the property that if one bacterium bound to any of the anti‐*Xf* in a given domain, the entire domain changed its work function due to a propagation effect.^[^ ^21^ ^]^ According to this model, a suitable function to fit the experimental dose‐response curves in Figure 2a,b was the 4‐Parameter Logistic curve,^[^ ^21^, ^39^, ^40^ ^]^ $\Delta I/I_{0}=A_{2}\;+\left(A_{1}-A_{2}\right)/\left[1+{\left(x/x_{0}\right)}^{p}\right]$, where *ΔI/I*_0_ is the SiMoT response, *x* is the nominal number of bacteria in 1 mL, *A_1_* and *A_2_* are the minimum and maximum response of the curve, defining the assay dynamic range;^[^ ^41^ ^]^ *x*_0_ is the inflection point where the curvature changes sign marking the analyte concentration where a response decrease of 50% occurs.^[^ ^42^ ^]^ Moreover, *p* parameter controls the symmetry of the distribution. Remarkably, *x*_0_ and *p* fitting parameters could be correlated to the average $\bar{x}$ and the variance *σ* of the distribution of the number of anti‐*Xf* comprised in each domain,^[^ ^21^ ^]^ being $\bar{x}$ = *x*_0_ *Vpn* and *σ* =$\sqrt{{\bar{x}}^{2}}/p$, considering a detecting interface hosting a compact biolayer uniformly covering the gate area with a number *n* = 7.2 × 10^10^ of capturing antibodies and an incubation volume *V* of 100 µL. Remarkably, the average $\bar{x}$ and the variance *σ* of the distribution of the number of anti‐*Xf* comprised in each domain had been estimated as $\bar{x}$ = 4.2 × 10^10^ and *σ* = 4.3 × 10^10^. Moreover, the average $\bar{x}$ and the variance *σ* of the distribution of the number of anti‐*Xf* comprised in each domain for the bacteria assay in sap, had been estimated as $\bar{x}$ = 6.5 × 10^10^ and *σ* = 6.6 × 10^10^. Those values very well compare with the average $\bar{x}$ and the variance *σ* of the distribution of the number of anti‐IgG comprised in each domain of a SiMoT sensing gate detecting IgG at the single‐molecule level.

A summary of all parameters of the logistic function (*A_1_, A_2_, x*_0_ and *p*) for the dose‐response curves registered in PBS and in sap, is reported in the **Table** **1** below.

**Table 1:** Logistig function fitting parameters used to model the dose‐response curves registered in PBS and in sap

4PL parameter	Dose response in PBS	Dose response in sap
*A*_1_ = [−Δ*I*/*I*_0_]^m^ *^i^* ^n^	0.07 ± 0.01	0.01 ± 0.03
*A*_2_ = [−Δ*I*/*I*_0_]^max^	0.68 ± 0.01	0.74 ± 0.02
*x*_0_	44 ± 14 bacteria per mL	67 ± 21 bacteria per mL
*p*	0.97 ± 0.2	0.97 ± 0.1

### qPCR Assay

Quantitative PCR was carried out on a CFX96TM Real‐Time System (Bio‐Rad Laboratories, USA) using Maxwell RSC PureFood GMO and Authentication Kit (Promega) according to manufacturer's protocol.^[^ ^21^ ^]^ The qPCR cycle number at which fluorescence reached a threshold value of nine times the standard deviation of baseline fluorescence emission was used for quantitative measurement.^[^ ^43^ ^]^ This being equivalent, in terms of the statistical level of confidence, to the LOQ estimated in the SiMoT assay. This cycle number was called the quantification cycle (*C*_q_) and it is inversely proportional to the starting amount of target genetic material. In particular, the *C*_q_ values, measured in triplicate for each assayed sample, showed a linear correlation with the bacterial concentrations for the samples containing from 10^6^ to 10^2^ bacteria per mL. In this range, a ∆*C*_q_ of 3 between two subsequent standard solutions had been registered. On the contrary, qPCR reactions on the samples containing 10 bacteria per mL of *X. fastidiosa* cells, yielded inconsistent results between the replicates and/or *C*_q_ values very close to those recorded for the previous dilution (10^2^ bacteria per mL). According to the measured *C*_q_ values, the samples were categorized as negative (*C*_q_ > 35), positive (*C*_q_ 20–33) or undetermined (*C*_q_ 33–35).^[^ ^24^ ^]^

## Conflict of Interest

The authors declare no conflict of interest.

## Data Availability

The data that support the findings of this study are openly available in IDA service at https://ida.fairdata.fi/login, reference number 101040383.
